# Making Sense of Proprioception by Bibliometric Research

**DOI:** 10.1002/brb3.70610

**Published:** 2025-06-17

**Authors:** Kevser Sevik Kacmaz

**Affiliations:** ^1^ Department of Physical Therapy and Rehabilitation Izmir Katip Celebi University Cigli Izmir Turkey

**Keywords:** bibliometric, proprioception, rehabilitation, scientific mapping, scientometric

## Abstract

**Background:**

Proprioception is one of the most significant factors in balance, stability, fine movements, coordination, and injury prevention. Proprioception research helps clarify how the nervous system integrates sensory inputs to plan and execute movements. Bibliometric analyses offer a systematic and comprehensive understanding of a field's structure, evolution, trends, research clusters, and gaps, laying a scientific foundation for future research. This study employs bibliometric analysis to provide a panoramic view of proprioception research and to identify its thematic structure, evolution, production, and impact.

**Methods:**

A total of 4506 original studies from 1979 to 2024 were extracted from the WoS. Using the Bibliometrix application in RStudio, a bibliometric analysis examined scientific performance, production, citation impact, research trends, developments, and the conceptual framework related to proprioception research. The Biblioshiny application performed the scientific mapping.

**Results:**

Proprioception research has increased linearly. The most influential article was Sensorimotor System Measurement Techniques, published in the Journal of Athletic Training, with 455 citations. Uwe Proske was the most influential author, with an *h*‐index of 20 in proprioception. The literature utilized 6797 keywords. Of these, 29% was proprioception, 4% joint position sense, and 4% rehabilitation. Keyword trends showed a shift toward rehabilitation and neurophysiology, with terms such as “rehabilitation,” “balance,” and “stroke” becoming more prevalent. However, an emerging interest in psychophysics, which investigates the interaction between proprioception and sensory perception, is also evident. This theme offers significant opportunities for future research. The USA leads in productivity, contributing 57.70% of the total publications, followed by Canada with 19.32%, and the UK with 18.28%.

**Conclusions:**

The results indicate a significant upward trend in research output, highlighting the increasing importance of proprioception in clinical and research settings. The findings emphasize several gaps in current proprioception research, including the need for greater interdisciplinary collaboration, particularly with neuroprosthetics and AI‐driven proprioceptive modeling. Furthermore, geographical diversity in research, particularly from underrepresented regions, is critical for comprehensively understanding proprioception across diverse populations. This study provides actionable information for researchers, clinicians, and policymakers. It urges future investigations to address these gaps and explore innovative approaches to enhance proprioception‐based therapies and technologies.

## Graphical abstract

Proprioception research is dynamic and seems likely to continue growing. Research on proprioception is increasingly prevalent in neuroscience, neurophysiology, and rehabilitation.

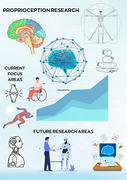

## 1 Introduction

Proprioception, broadly described as the sense of body position and movement, is crucial in motor control, spatial orientation, and various neuromuscular processes (Héroux et al. 2022; Moon et al. 2021). Its dysfunction is linked to numerous conditions, including orthopedic injuries, neurological disorders, and age‐related deficits, that can cause motor function disorders and decreased quality of life (Sevik Kacmaz and Unver 2023; Röijezon et al. 2015). Accordingly, understanding proprioception is pivotal in rehabilitation, sports medicine, neurology, and neuroscience.

Despite more than a century of empirical work, proprioception research remains conceptually diffuse and methodologically heterogeneous. Key constructs—joint‑position sense, kinesthesia, force sensing, efference copy—are often operationalized differently across studies, making cross‑trial synthesis difficult and, at times, statistically inadmissible (Proske and Gandevia 2012). Assessment protocols vary from psychophysical threshold tests and tendon‑vibration paradigms to instrumented joint‑reposition tasks and neuro‑imaging surrogates, each capturing only a subset of the underlying multisensory construct. Inter‑laboratory differences in stimulus velocity, movement amplitude, reference angle, and limb dominance further erode comparability (Goble 2010). At the clinical level, outcome measures lack consensus minimal‑detectable‑change values, hampering meta‑analytic aggregation and evidence‑based guideline development (Hillier et al. 2015). Finally, disciplinary differences persist. Neuroscientists focus on central mechanisms, sports scientists on injury prevention, and rehabilitation researchers on functional recovery—often without shared taxonomies or interoperable datasets. These conceptual and methodological fault lines highlight an urgent need for unified frameworks, standardized outcome sets, and cross‑disciplinary consortia to consolidate the evidence base and accelerate translational impact. However, advances in genetic tools for proprioceptive circuits now offer new opportunities to explore the central pathways involved in the proprioceptive process (Marasco and de Nooij 2023). Advancements in targeting neurons are swiftly improving our capacity to manipulate proprioceptive and motor circuits (Dallmann et al. 2021). Simultaneously, new data from emerging neural‐machine interface technologies can modulate the sense of agency and provide insights into the proprioceptive mechanisms (Marasco and de Nooij 2023). Advances in prosthetic limbs, brain–body interfaces, and artificial intelligence (AI) have fueled research into brain–body interactions, focusing on movement control, spatial orientation, and perception (Hagert and Rein 2024; Xue et al. 2022). Such studies are also relevant as robotic technologies shift toward more automated processes, where versatile robots that can perform various human movements are developed with proprioception provided by inertial measurement units (Xue et al. 2022; Kwiatkowski et al. 2017).

With the rapid rise in scientific production, keeping up‐to‐date scientific evidence and practice is becoming increasingly difficult (Turgut and Beyaz 2024). While traditional literature reviews and meta‐analyses have significantly contributed to synthesizing knowledge on proprioception, these approaches often focus on a narrow set of hypotheses or a limited range of study designs (Kumar et al. 2023). In contrast, bibliometric analysis offers a systematic and comprehensive overview of an entire research domain, revealing patterns in authorship, publishing trends, citation networks, and keyword co‐occurrences. This panoramic view helps capture the breadth and depth of proprioception research, from core themes to emerging areas (Öztürk et al. 2024). In addition, bibliometric tools enable mapping conceptual structures and thematic clusters, providing insights that traditional reviews or meta‐analyses may overlook, especially when evaluating interdisciplinary fields (Carballo‐Costa et al. 2022; Aria and Cuccurullo 2017). Hence, bibliometric analysis is particularly advantageous in this study for uncovering the global research landscape, trends, and collaborative networks that have shaped—and continue to shape—proprioception research.

Despite these strengths, it is also critical to acknowledge potential methodological biases and limitations inherent in bibliometric analyses. For instance, the reliance on citation‐based metrics can be influenced by the “Matthew effect,” wherein already well‐cited papers garner even more citations due to their perceived prestige rather than their intrinsic scientific merit (Hirsch 2007). Citation counts may also reflect factors such as journal reputation or self‐citations rather than strictly denoting scientific quality (Bornmann and Daniel 2007; Hirsch 2005). Moreover, the choice of database inevitably excludes studies indexed elsewhere, thus introducing selection bias (Tang et al. 2024). Language bias further arises when non‐English publications remain underrepresented, and certain regions or institutions may disproportionately shape citation networks based on their research funding and visibility (Waltman 2016). Consequently, while bibliometric methods excel at revealing macro‐level patterns, researchers should interpret citation‐based impact measures with caution and consider triangulating findings with other review methodologies where feasible.

Despite the growing interest in proprioception, systematic bibliometric analyses are lacking. Only one study has used bibliometric methods to examine proprioception. However, it has a limited scope since it only investigated the proprioception literature on gymnastics research (Fallas‐Campos et al. 2023). Other than that, no systematic bibliometric study has investigated proprioception research. Proprioception is one of the most significant factors in gross and fine motor tasks, joint stability, balance, coordination, and injury prevention. Proprioception research helps identify how the nervous system integrates sensory afferents to plan and execute movements. Therefore, it is critical for proprioception research to develop and get deeper. Clarifying core themes, research gaps, emerging and declining trends, productivity, and the impact of the various factors helps understand the research area and improve proprioception research further. Therefore, by identifying thematic clusters, tracing the field's historical development, and highlighting emerging trends, we aim to provide valuable insights for researchers, clinicians, and policymakers who seek to advance both the science and practice of proprioception in diverse healthcare settings.

## 2 Materials and Methods

### 2.1 Search Strategy and Screening

The literature search was conducted on August 1, 2024 in the Web of Science Core Collection (WoS) to identify articles from inception to August 1, 2024.

The Boolean search strategy was ‘’propriocept* OR “joint position sense” OR kinesthesis* OR “joint position*” OR “position sense” OR “movement sensation” OR “sense of position” OR “joint motion” OR “movement sense” OR “joint reposition*” OR “motion threshold” OR “movement threshold” OR “motion perception” in the title search and revealed 8539 publications. The systematic reviews, reviews, meta‐analyses, and key publications in the field were examined to determine the search terms, and trial and error determined that these are the most comprehensive keywords. In addition, different combinations were tried with codes such as ‘or, and, *,’…’ (Héroux et al. 2022; Moon et al. 2021; Röijezon et al. 2015; Fallas‐Campos et al. 2023; Zupic and Čater 2015; Kaçmaz and Çelik 2025). An independent expert researcher reviewed and validated the research method (Zupic and Čater 2015; Kaçmaz and Çelik 2025).

We applied filters to include only peer‐reviewed, original research by designating the publication type as either research articles or reviews, resulting in 5988 articles. After filtering by the English language, we retained 5817 articles. This analysis was planned to report on human health‐related evidence. Therefore, we filtered the research fields of the publications to focus solely on research related to human health sciences, such as physical therapy and rehabilitation, surgery, neuroscience, and others, which left us with 4506 articles.

After downloading the data, we completed the missing data by checking the authors, countries, journals, and keywords, preparing them for analyses (Sabancı Baransel et al. 2023). Since only one database was used and no data was merged, there was no duplicate data.

Retracted articles were checked using Koo and Lin (2024) method. A specific feature identifies retracted articles in WoS. This tool makes it easier to quickly identify and eliminate retracted papers. However, there was no retracted article in our dataset.

### 2.2 Data Analysis

The data, including title, author, publication year, journal, country, citation count, keywords, field of study, *h*, *g*, *m* indexes, and the journal publication years, were extracted (Li et al. 2023). The obtained data were downloaded in plaintext format, exported into Microsoft Excel (Microsoft Corporation, USA), and presented in frequencies and percentages.

Bibliometric studies can be conducted using a variety of databases. Nevertheless, several databases have restrictions and issues when used with other databases. For instance, while many of these databases include bibliometric data with a more constrained scope, some do not permit downloading bibliometric data. Furthermore, when merging data sets, presenting the data in disparate formats and a different sequence causes technical issues, data loss, and data overlap, which cast doubt on the conclusions. Therefore, working on a single database is recommended (Öztürk et al. 2024; Kaçmaz and Çelik 2025).

The reasons for selecting the WoS database are that it includes a significant portion of high‐quality research and top‐impact journals, it provides a comprehensive citation‐searching mechanism that spans various topics disciplines (Bang et al. 2019), and it is the preferred database for bibliometric analyses because of its rigorous evaluation process and reliable, high‐impact information (Bang et al. 2019; Kaçmaz et al. 2024), which will allow researchers to compare and interpret results of other studies.

### 2.3 Bibliometric Analysis

Bibliometric analyses involved performance analysis and scientific mapping.

The Bibliometrix package v. 4.3.0 in RStudio (R Core Team, Austria) performed the performance evaluation and scientific mapping (Aria and Cuccurullo 2017). The Biblioshiny web interface provider v. 4.1.3 visualized the analysis outputs.

Objective performance analysis examines the contribution of research components, while scientific mapping shows the associations between research components (Donthu et al. 2021; Çelik and Karaca 2024). Trends and hotspots in research can be identified by analyzing the most frequent keywords and their evolution over time (Tsiamalou et al. 2022).

The performance analyses examined the quantitative characteristics of the literature. The number of articles and citations is the most fundamental performance measure representing productivity, impact, or influence (Ma et al. 2024; J.‐W. Chen et al. 2022). Citation analysis measures an author, journal, or article's impact on a field (Khatra et al. 2021). Productivity and impact were analyzed by year, journal, author, citation, and country. For multi‐country studies, the country of the corresponding author was considered. Local citations refer to the citations made by the studies in the dataset, whereas global citations encompass all citations within the WoS (Kaçmaz et al. 2024).

We conducted analyses based on three basic bibliometric indicators: *h*‐index, *g*‐index, and *m*‐index, which are widely used in the literature. *h*‐index, as a measure of an author's citation and productivity balance, is based on the condition that the author has an “h” number of publications with at least “h” citations (Hirsch 2005). The *g*‐index is an indicator that highlights highly cited publications relatively more, making the impact of the most cited studies more prominent in the distribution of “total citations” (Egghe 2006). *m*‐index relates the author's *h*‐index to the duration of their academic career, which is intended to provide a more comparable profile, especially for early career researchers (Hirsch 2005).

The source impact and Bradford's Law analyses were also conducted to identify the primary journals (Bozkurt and Bozkurt 2024). Bradford's law suggests that a core group of journals publishes one‐third of the articles on a topic, making them the most influential. One‐third of the articles appear in *n* times the number of core journals, while one‐third *n* ^2^ times the number of core journals are published. The journals in the second group are considered less effective, while those in the third group are deemed ineffective. The core journals were determined according to the Leimkuhler distribution. All journals are sorted in descending order by the number of related papers they contain. A running cumulative total of articles is calculated. The ranked list is partitioned into three successive zones, each intended to include approximately one‐third of the articles (Leimkuhler 1967).

### 2.4 Scientific Mapping

Scientific mapping reveals the relationships between research components (Donthu et al. 2021; Bozkurt and Bozkurt 2024). In addition to being visualizations, these maps also serve as a serialized data pedigree, showcasing various complex associations such as networks, structure, evolution, or derivation among data units (Li et al. 2023). The most used and co‐occurred keywords were investigated to reveal the main study themes, trends, and conceptual structures (J.‐W. Chen et al. 2022). The size of words in word clouds increases based on the frequency of their occurrence. Keywords frequently appearing together are displayed as clusters (Ma et al. 2024). After removing empty terms (keywords already used in the Boolean operators), the 50 most frequently used keywords were visualized. In addition, thesaurus files were created to identify synonymous terms in the keyword analysis.

A strategic diagram, thematic map, or thematic analysis illustrates a field's evolution, trends, and developments. It employs clusters of keywords and their connections to highlight research themes. This provides insights for researchers and stakeholders about the potential for upcoming research within a field (Agbo et al. 2021). It has coordinates in two dimensions constructed from centrality and density. The *X*‐axis indicates centrality, showing the degree of domain coupling derived from the strength of ties between the topics in clusters. The *Y*‐axis represents density, indicating the strength of a field's internal connections and estimating the number of concurrent occurrences of each term (J.‐W. Chen et al. 2022; Agbo et al. 2021; Kacmaz and Kaçmaz 2024). The graph is divided into four quadrants, each illustrating the importance and development of a theme. Themes located in the lower left are either emerging or declining. In contrast, core themes are found in the lower right, indicating high centrality but low intensity, suggesting they have been extensively studied in the past. The upper left showcases isolated yet highly developed themes characterized by low centrality and high intensity. The upper right features basic and advanced motor themes (Bozkurt and Bozkurt 2024; Ahmi 2021).

This research was ensured to be reliable and valid by choosing a reliable and comprehensive database, predetermining concise inclusion and exclusion criteria, and using reliable software such as Bibliometrix and Biblioshiny (Bozkurt and Bozkurt 2024; J. Chen and Chang 2022). In addition, the bibliometric analyses calculate the authors, publication years, keywords, countries, and so on, such as Bradford's law analysis (Leimkuhler 1967). These data are objective and independent of the researchers. Therefore, each bibliometric analysis has a high level of sensitivity.

## 3 Results

### 3.1 Performance Analyses

**FIGURE 1:**
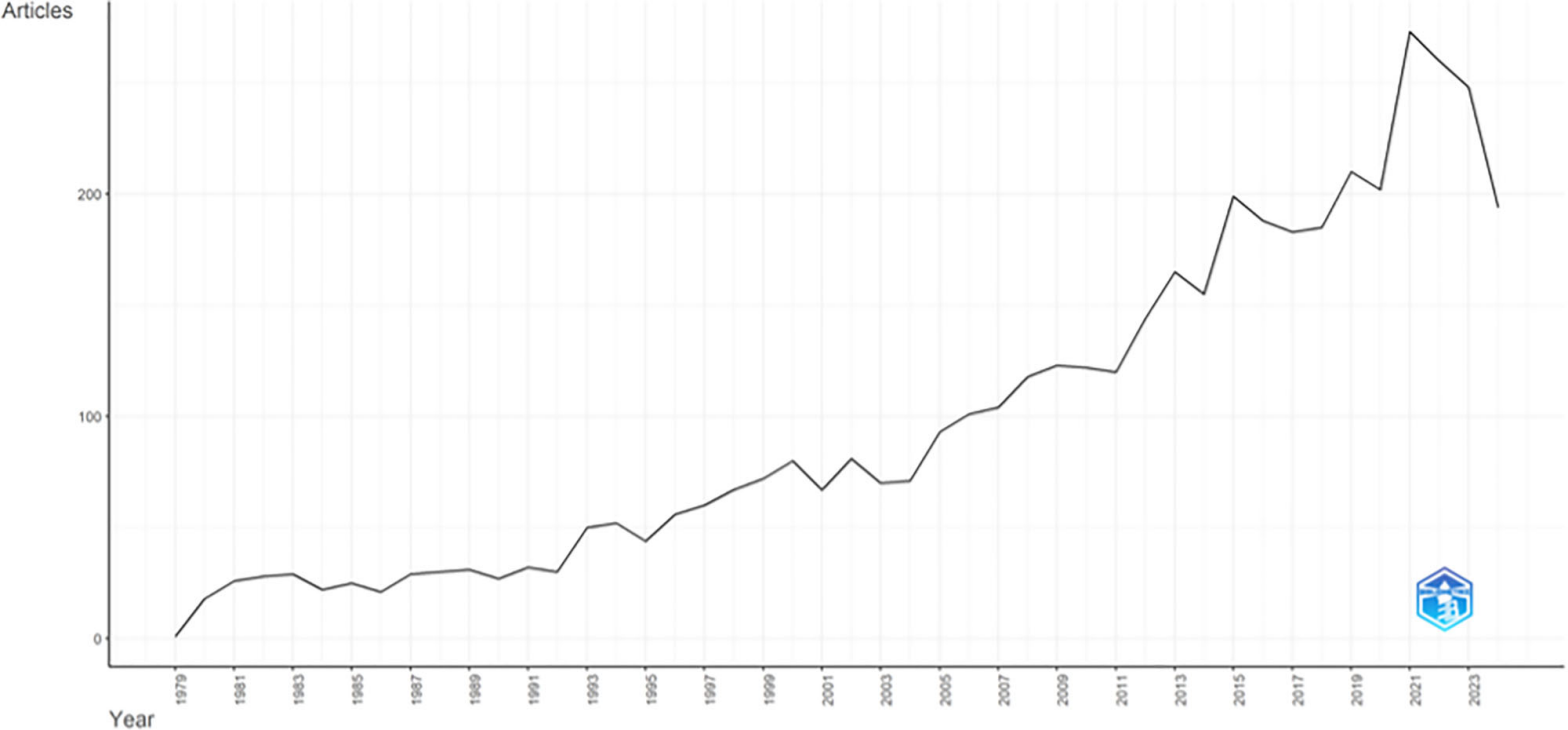
The annual publication numbers and global trends in proprioception.

From 1979 to 2024, there were 4506 articles, 4209 of which were research articles, and 267 were reviews. The year 2021 saw significant breakthroughs, resulting in 273 articles. Overall, the number of articles increased linearly, with a mean of 97.95 published yearly. The annual growth rate of publications was 12.42%. Figure 1 shows annual publication numbers in proprioception. It indicates a steady increase since the early 2000s and a rapid acceleration after 2020. This rise can be an important indicator of increasing interest in the field and multidisciplinary studies. The mean age of the articles was 13.1 years, and citations per article were 31.38.

### 3.2 Author Productivity and Impact

A total of 14,017 authors contributed to the literature, averaging 4.4 authors per article. A total of 210 authors published 248 articles individually. The co‐authorship rate was 21.77%.

The most productive researchers were Adams R, Waddington G, Han J, Proske U, Henriques DYP, Gandevia SC, Lephart SM, Cressman EK, Witchalls J, and Reddy RS. The most influential researchers are Proske U, Henriques DYP, Lephart SM, Gandevia SC, Goble DJ, Cressman EK, Roll JP, Adams R, Brumagne S, and Konczak J. Roger Adams was the most productive author, with 39 articles and an *h*‐index of 13 in proprioception. Uwe Proske was the most influential author, with an *h*‐index of 20 in proprioception.

### 3.3 Country Productivity and Impact

Seventy‐eight countries contributed to the literature, with the USA publishing more than half (57.70%), Canada with 19.32%, and the UK with 18.28%. The most productive countries include the USA, Canada, the UK, China, Italy, Germany, Japan, France, Australia, and South Korea.

**FIGURE 2:**
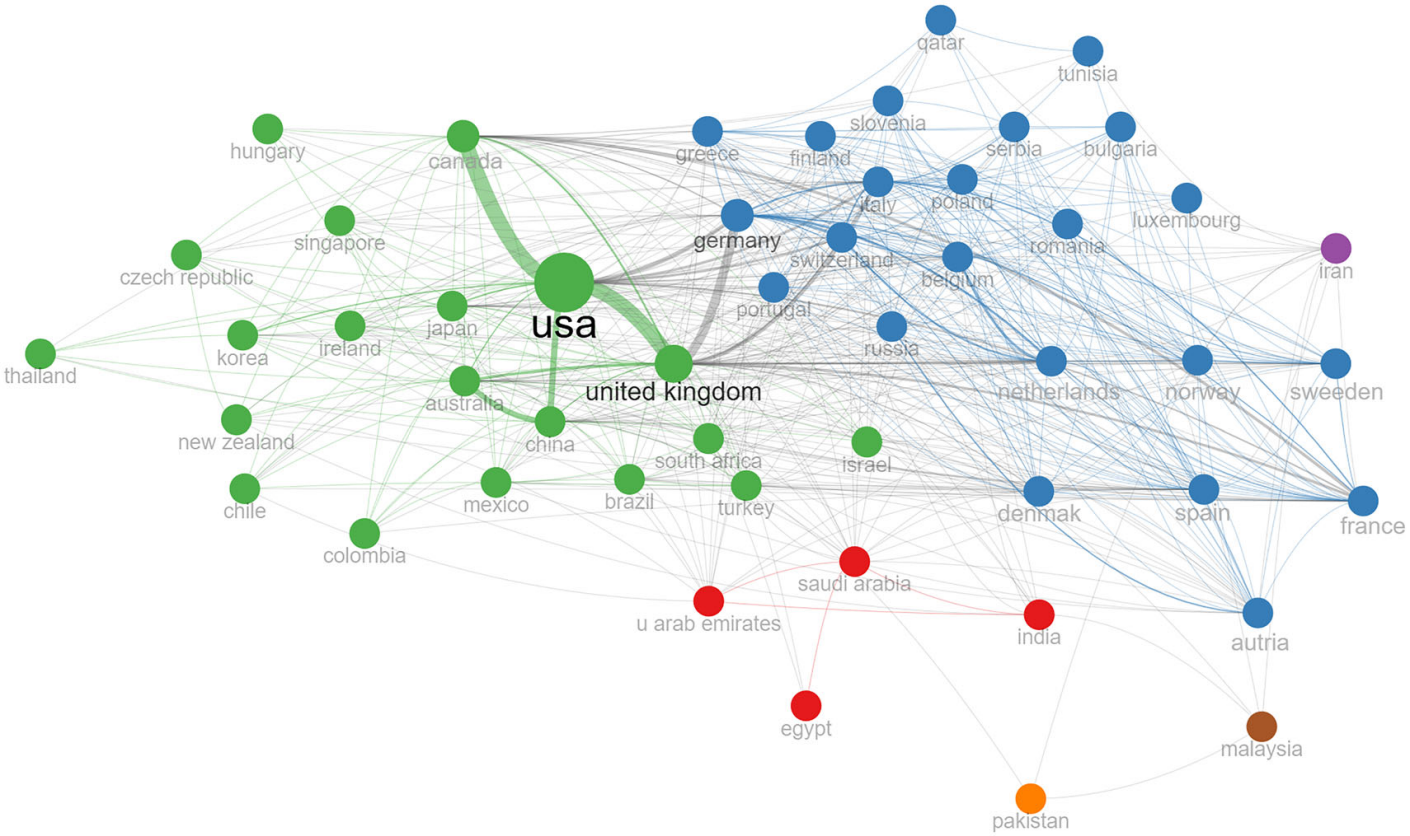
The collaborations between countries.

Figure 2 presents the collaborations between countries. The USA, Canada, the UK, and some European countries cooperate with each other more intensively than the rest of the world. Other European countries, such as Spain, Germany, Romania, Serbia, and Poland, have their own collaboration web, mostly with Eastern European countries. There is another small collaboration of four countries, including Egypt, India, the United Arab Emirates, and Saudi Arabia, in red nodes. Some countries, such as Iran, Malaysia, and Pakistan, stand alone. This shows that these countries are not yet fully integrated into cooperation networks.

### 3.4 Journal Productivity and Impact

The articles were published in 901 journals. The most productive journals on proprioception are Experimental Brain Research, Journal of Neurophysiology, Vision Research, Journal of Neuroscience, Journal of Physical Therapy Science, Journal of Vision, Neuroscience Letters, Neuroscience, Frontiers in Human Neuroscience, and Journal of Sport Rehabilitation.

Table 1 presents the most influential journals: Experimental Brain Research, Journal of Neurophysiology, Vision Research, Journal of Neuroscience, Journal of Physiology‐London, Journal of Athletic Training, Journal of Orthopaedic & Sports Physical Therapy, Archives of Physical Medicine and Rehabilitation, American Journal of Sports Medicine, and Neuropsychologia.

**TABLE 1:** The most influential journals.

Journal	*h*_index	*g*_index	*m*_index	Citation (number)	Article (number)	PY_start
Experimental Brain Research	52	83	1.156	9430	246	1980
Journal of Neurophysiology	45	83	1.047	7475	146	1982
Vision Research	40	62	0.889	4438	117	1980
Journal of Neuroscience	39	69	0.929	6202	69	1983
Journal of Physiology‐London	28	41	0.622	2682	41	1980
Journal of Athletic Training	27	36	0.964	2518	36	1997
Journal of Orthopaedic & Sports Physical Therapy	26	32	0.839	2294	32	1994
Archives of Physical Medicine and Rehabilitation	25	42	0.581	2226	42	1982
American Journal of Sports Medicine	21	25	0.512	2777	25	1984
Neuropsychologia	21	34	0.618	1225	42	1991

*Note*: PY_start: The year of starting to publication.

According to Bradford's law, there were 25 core journals and 876 non‐core journals. These core journals accounted for 2.77% of the total publications and 33.66% of the total articles.

Table 2 highlights the most influential articles. Experimental neurophysiology topics dominate the proprioception literature, with proprioceptive alterations in various knee conditions closely following.

**TABLE 2:** The most influential articles.

Article title	Journal	Author(s)	Year	Local citations	Global citations
Sensorimotor system measurement techniques	*Journal of Athletic Training*	Riemann BL et al.	2002	349	455
The proprioceptive senses: Their roles in signaling body shape, body position and movement, and muscle force	*Physiological Reviews*	Proske U et al.	2012	300	1115
Joint proprioception in normal, osteoarthritic and replaced knees	*The Journal of Bone & Joint Surgery British Volume*	Barrett DS et al.	1991	151	434
The role of proprioception in the management and rehabilitation of athletic injuries	*American Journal of Sports Medicine*	Lephart SM et al.	1997	150	476
Proprioceptive Acuity Assessment Via Joint Position Matching: From Basic Science to General Practice	*Physical Therapy*	Goble DJ	2010	127	235
Alteration of proprioceptive messages induced by tendon vibration in man: A microneurographic study	*Experimental Brain Research*	Roll JP et al.	1989	120	660
Proprioception in the anterior cruciate deficient knee	*American Journal of Sports Medicine*	Barrack RL et al.	1989	111	356
Age‐related decline in proprioception	*Clinical Orthopaedics and Related Research*	Skinner HB et al.	1984	108	367
A selective impairment of motion perception following lesions of the middle temporal visual area (MT)	*Journal of Neuroscience*	Newsome WT et al.	1988	99	1247
Proprioception and function after anterior cruciate reconstruction	*The Journal of Bone & Joint Surgery British Volume*	Barrett DS	1991	98	295

### 3.5 Scientific Mapping

#### 3.5.1 The Most Used and Co‐Occurring Keywords

**FIGURE 3:**
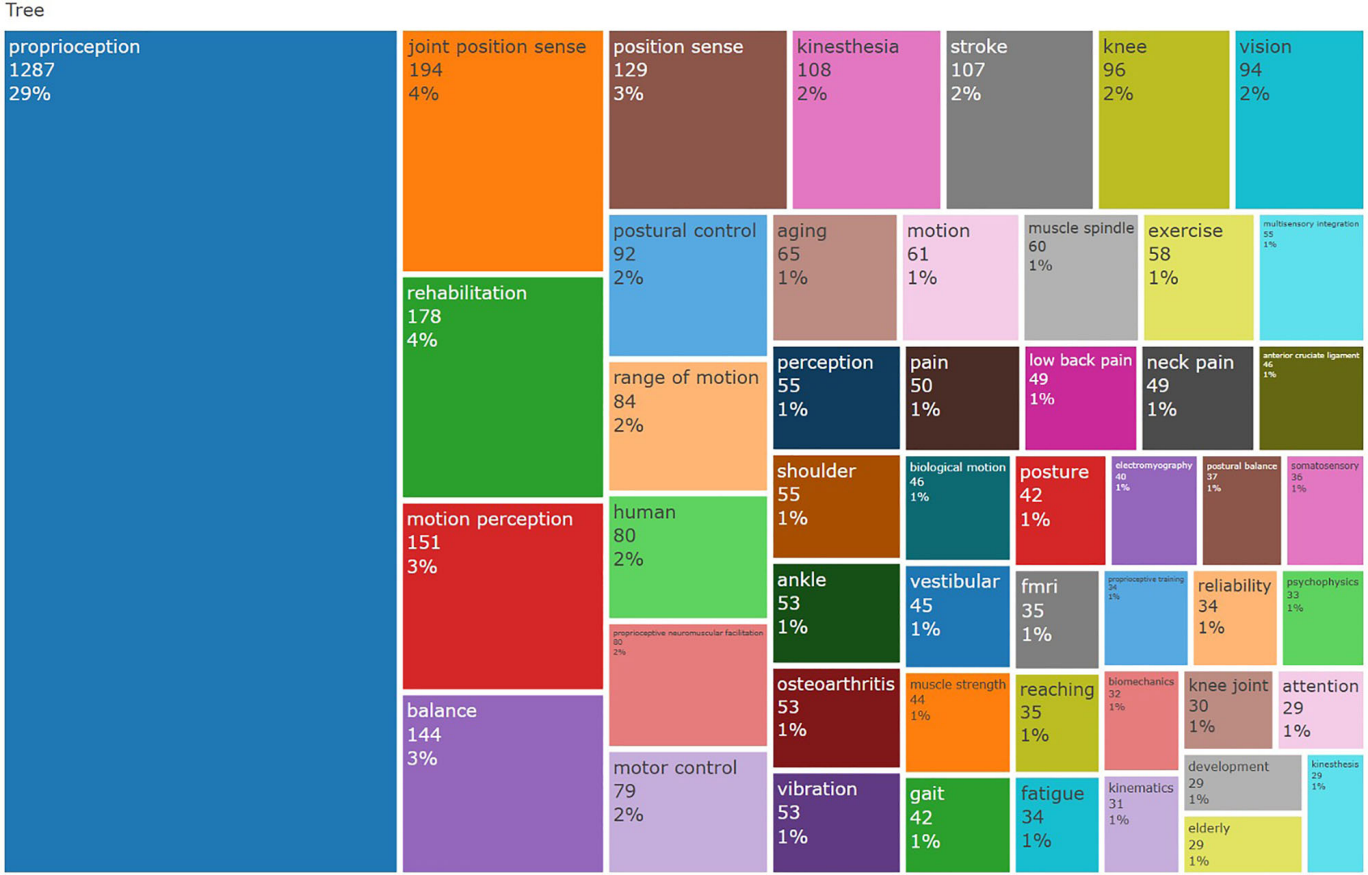
The most used keywords.

The literature utilized 6797 keywords. The most frequently used keywords include rehabilitation, balance, stroke, knee, vision, postural control, range of motion, human, proprioceptive neuromuscular facilitation, and motor control. Figure 3 shows the most common keywords with their percentage of usage. Of these, 29% was proprioception, 4% was joint position sense, and 4% was rehabilitation. The keywords “rehabilitation” and “balance” are prominent, whereas “motion perception” and similar themes are less frequent. This trend suggests proprioception research is moving toward more clinical and applied aspects.

**FIGURE 4:**
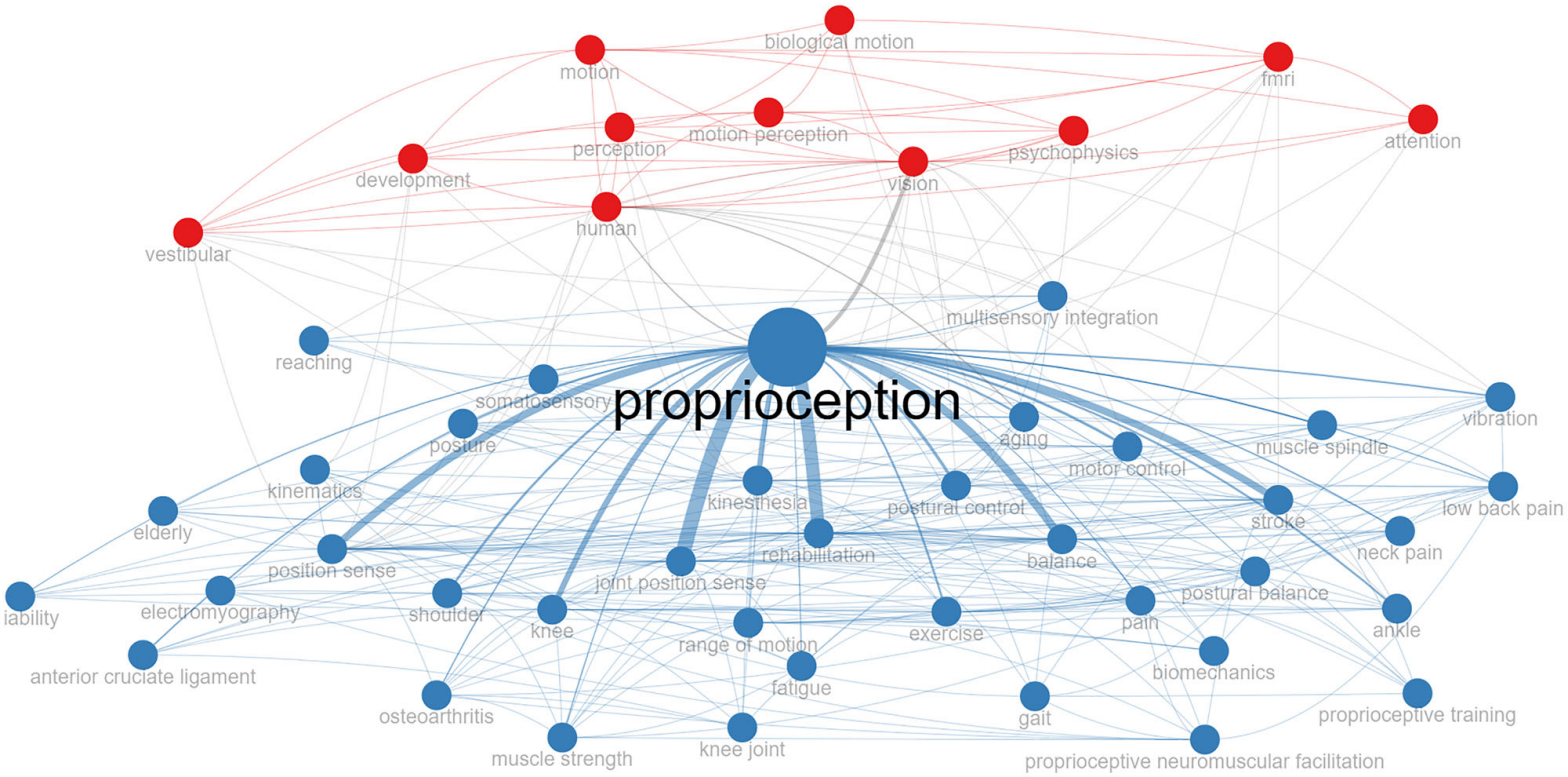
The co‐occurrence analysis of the keywords.

The co‐occurrence analysis revealed two distinct clusters (Figure 4). In the blue cluster, terms such as “rehabilitation,” “stroke,” and “postural control” are heavily associated. In the red cluster, the co‐occurrence of the keywords “vision,” “perception,” and “psychophysics” reveals studies linking proprioception to perceptual processes and cognitive mechanisms. This distinction shows the separation between the different foci of the field (clinical practice and basic science).

**FIGURE 5:**
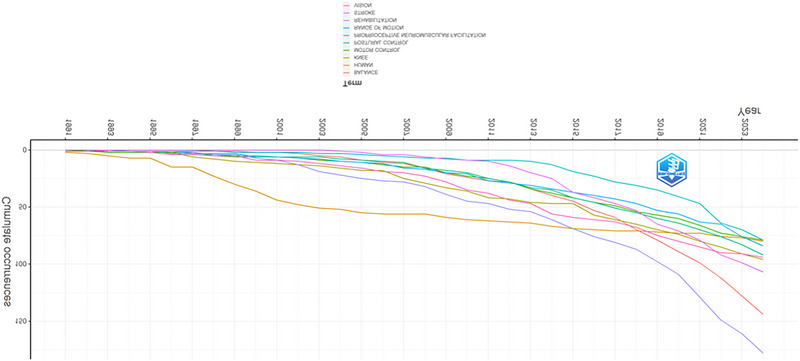
The dynamic analysis of keywords.

Figure 5 presents a dynamic analysis of keyword use frequency in proprioception over time. The horizontal axis shows the years (1984–2023), and the vertical axis shows the cumulative co‐occurrence values.

Although the frequency of all keywords has consistently increased over the years, rehabilitation has seen a significant rise, becoming the keyword with the highest frequency of use. There is a shift from general terms (e.g., “movement,” “human”) to more specific and technical terms (e.g., “sensorimotor control,” “postural control”). In clinical and applied contexts (e.g., “knee,” “balance”), we see the words persist or gain in importance. This change shows that the field is maturing and that the focus is now more on application areas than basic concepts.

**FIGURE 6:**
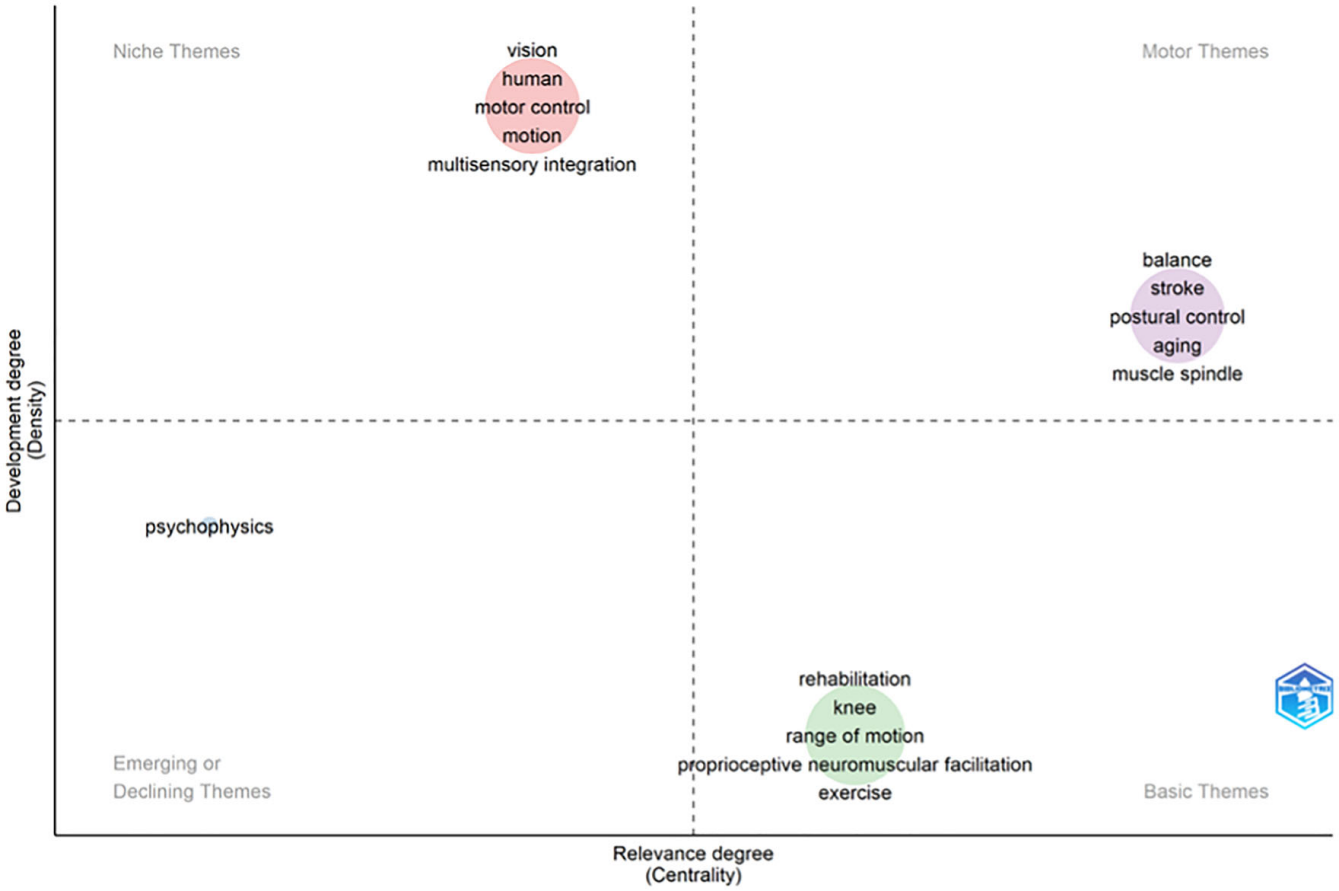
The strategic diagram of the keywords.

Figure 6 shows the strategic diagram. Cluster 1 includes balance, stroke, postural control, aging, and muscle spindle. Cluster 2 comprises vision, human, motor control, motion, and multisensory integration. Cluster 3 encompasses psychophysics. Cluster 4 includes rehabilitation, knee, range of motion, proprioceptive neuromuscular facilitation, and exercise. Thus, rehabilitation‐centered research appears to have the most significant prevalence and impact in proprioception.

**FIGURE 7:**
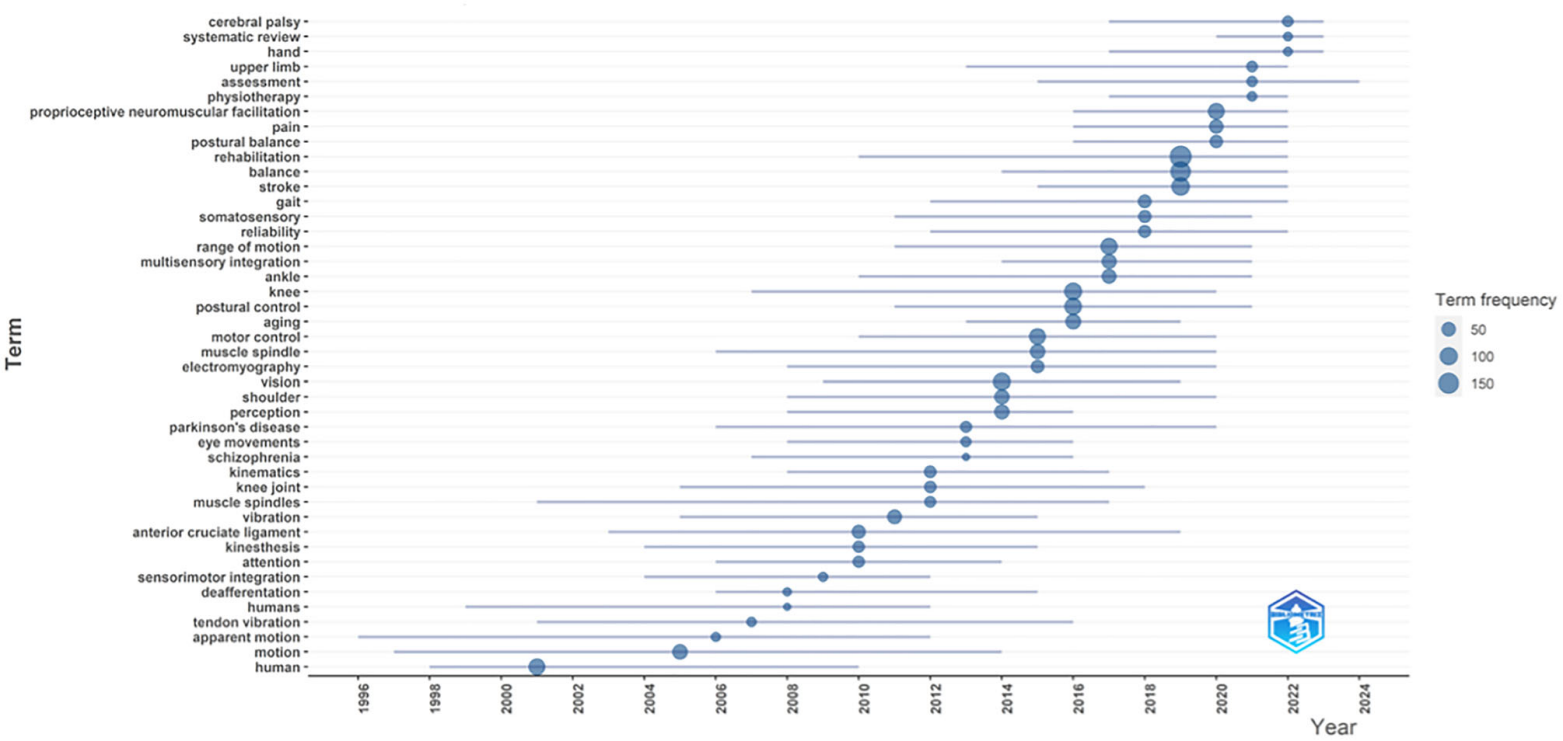
The trend topics.

Figure 7 presents the distribution of the most frequently used keywords over time. The horizontal lines represent the evolutionary process, while the size of the dots indicates keyword frequency. In 2023, cerebral palsy (*f* = 24), hand (*f* = 17), systematic review (*f* = 17) is trending; in 2022, upper limb (*f* = 25), assessment (*f* = 23), physiotherapy (*f* = 19) are trending topics; and in 2021, proprioceptive neuromuscular facilitation (*f* = 79), pain (*f* = 50), postural balance (*f* = 37) are notable for their trend status. “Motion” and “muscle spindles” had the longest durations.

## 4 Discussion

This study employed bibliometric analyses to investigate proprioception research. The research has gradually increased and is expected to expand, with a current surge in publications showcasing heightened scientific productivity. Research focuses on rehabilitation and neuroscience/neurophysiology themes, particularly emphasizing rehabilitation, balance, and stroke, with increasing attention to psychophysics. Following various pathologies, the knee joint has been the most studied joint in proprioception. This focus is particularly evident in research conducted by orthopedics and rehabilitation disciplines, as seen in other analyses within this study.

The overall publication trend is upward with minor fluctuations. In 2021, studies achieved significant breakthroughs, suggesting that the increased attention on proprioception may stem from a deeper understanding of its relevance. The annual growth rate is 12.42%, indicating the research field is dynamic and expanding. The field has not yet reached saturation and remains open to various developments. This rise in production emphasizes the growing interest in proprioception to enhance the efficiency of clinical and research practices and the rising focus on preventive healthcare (Tang et al. 2024). These results align with similar studies by Kokol et al. (2021) and Bartik et al. (2024), showing a comparable growth trend in medicine.

### 4.1 Underlying Factors Influencing the Growth of Proprioception Research

This study observed that proprioception is growing rapidly, especially in rehabilitation, neurophysiology, and clinical applications. Several factors may explain this rise. First, technological advances—especially robotic prostheses, AI‐assisted devices, and neuroimaging techniques—have enabled researchers to study the mechanisms of proprioception using more sensitive methods (Xue et al. 2022). Second, policy changes and expanding rehabilitation services from a public health perspective have encouraged multidisciplinary teams to turn to proprioception‐based approaches (Maseko et al. 2024). For example, with an aging population and the increase in chronic diseases, developing more comprehensive rehabilitation policies for movement and function losses increases the importance of proprioception research. Third, as the role of proprioception in a wide range of conditions, from sports injuries to cerebrovascular diseases, has been emphasized, awareness has increased in both the clinical and academic communities (Proske and Gandevia 2012).

The combination of these factors suggests that the current growth trend in proprioception research is driven by scientific curiosity and societal and technological demands. By combining bibliometric analyses with qualitative assessments (e.g., expert surveys and field studies), future studies can increase the data's validity and holistically examine the field's development.

The citations highlight the attention and demand for proprioception research (J.‐W. Chen et al. 2022; Bartik et al. 2024). Highly cited literature usually indicates significant impact, providing valuable insights into critical focus areas. Analyzing the most cited research can offer early indications of emerging research trends and directions (Xu et al. 2024). The most cited paper was “Sensorimotor System Measurement Techniques,” published in the Journal of Athletic Training in 2002. It was cited 455 times globally and 345 times in proprioception studies, which could be interpreted as indicating it is the most influential paper containing core principles of proprioception assessment. The ten most influential papers were about 20–30 years old, with the newest being 14. Generally, it takes 10–20 years to achieve maximal recognition and influence regarding citations (Khatra et al. 2021). In addition, cumulative citations may favor older articles. In a bibliometric study, 54 out of 199 articles with 10,000+ citations were published before 1973, and 170 were published before 2000 (Liu et al. 2018). Therefore, the effects of time must be considered in the citation count.

### 4.2 Over‐Reliance on Citation‐Based Metrics and Bibliometric Limitations

An important issue to consider in interpreting bibliometric findings is citation bias. It can be misleading to assume that highly cited studies always have the most robust methodology, the highest clinical value, or impact (Bornmann and Daniel 2007; Waltman 2016). The data provided by citation‐based metrics do not directly reflect the actual level of scientific impact and societal benefit. For example, the high number of citations a particular article receives may be related to factors such as focusing on a popular topic, being published in a highly accessible journal, or having a large number of self‐citations, rather than the article being innovative or of high quality (Hirsch 2005). In addition, certain regions or disciplines with a major impact in the clinical or applied field may lag behind in citations because a limited academic audience reads it (Waltman 2016; Kraus et al. 2022).

Moreover, citation habits common in academic circles can lead to more frequent citations of work published in prestigious journals and institutions, creating a self‐reinforcing cycle called the Matthew effect around journals or institutions that tend to be highly cited (Hirsch 2005). This cycle can artificially increase the number of citations for some authors and institutions, making the true scientific impact appear higher than it actually is. In addition, some self‐citation or regional publication preferences reinforce citations from authors themselves, certain groups, or regions.

Our study's data is largely based on publications in high‐impact journals and English‐language articles prioritizing a specific global research ecosystem. This can make it difficult for publications in other languages or with lower impact factors to receive the citations they deserve. Therefore, although our bibliometric results largely reflect the overall dynamics of the field, it is clear that we should not ignore studies that are “invisible” due to citation bias. In future studies, it is important to reduce the effect of citation bias and make the analysis more inclusive by combining multiple databases or including journals published in local languages, if possible. Considering different metrics (e.g., altimetric, expert review panels, rate of inclusion in clinical guidelines, etc.) when evaluating the impact of studies on proprioception may offer a more balanced and inclusive perspective (Kraus et al. 2022). Future research could better reflect proprioception research's real‐world importance and uses by considering these alternative evaluation methods in conjunction with bibliometric results.

### 4.3 Country Productivity and Impact

Seventy‐eight countries contributed to the literature, indicating that proprioception has received significant attention and its importance has been well‐established. North America led the field of proprioception research, with the USA accounting for more than half of all publications, followed by Canada and the UK. This highlights the influence of financial and intellectual resources on scientific production and research capacity (Fallas‐Campos et al. 2023; H. Wang et al. 2022). This result is understandable because North America and Europe have numerous higher education institutions, high‐tech industries, and laboratories (Tang et al. 2024). Greater access to funding opportunities, financial resources, institutional collaborations, job opportunities in research fields, enhanced intellectual and educational capacity, active research environments, and collaborative teams may stimulate scientific production (J.‐W. Chen et al. 2022; Khatra et al. 2021). Furthermore, the uneven geographical distribution of research indicates that global diversity in this field has not yet been fully realized. This emphasizes the possible necessity for research to enhance geographical diversity, considering cross‐cultural variations and normative data.

In addition, the predominance of English‐language publications in databases such as WoS means that local research communities—for example, studies produced in different languages in Asia, South America, or the Middle East—are not adequately reflected in the literature (Waltman 2016). This shortcoming may lead to an underestimation of local/population‐based research data and a lack of geographical diversity, especially in areas with high clinical relevance, such as proprioception.

### 4.4 Author Productivity and Impact

A total of 14,017 authors contributed to the literature, averaging 4.4 authors per article. The co‐authorship rate is 21.77%. This high number of authors per article indicates that researchers in proprioception tend to publish their studies collaboratively (Bartik et al. 2024), pointing to the complexity and multidisciplinary nature of proprioception research. A total of 210 authors published 248 articles by themselves. These studies may be empiric physiological studies investigating various theories.

The most productive authors are Adams R, Waddington G, Han J, Proske U, Henriques DYP, Gandevia SC, Lephart SM, Cressman EK, Witchalls J, and Reddy RS. Proske U, Henriques DYP, Lephart SM, Gandevia SC, Goble DJ, Cressman EK, Roll JP, Adams R, Brumagne S, and Konczak J are the most influential authors. Barrett DS has two articles in the 10 most influential articles. The studies of these authors may shape and guide proprioception research by providing concepts and theoretical support. Following these, authors could effectively monitor research trends and new evidence (Xue et al. 2022).

### 4.5 Co‐Authorship Patterns and Collaborations

The overall co‐authorship rate was 21.77%. This indicates that researchers in proprioception tend to collaborate intensively. According to the analyses, inter‐country collaborations increase with geographical proximity. Closer countries collaborated more. The USA, Canada, and developed North Atlantic European countries are cooperating more with each other. The intensity of collaborations between these countries reflects the density of research funding, universities, and laboratory networks in these countries. Some countries, such as Iran, Malaysia, and Pakistan, are thinly delineated and isolated on the map. This means these countries have a relatively small share of the research literature or are not yet fully integrated into cooperation networks. More intensive cooperation with these regions in the future is a potential research area for increasing geographical diversity.

Researchers can facilitate rapid progress in the field by engaging with countries concentrated in specific clusters and developing joint papers and projects. Policymakers can organize calls for funding and joint projects to balance unbalanced collaboration networks and ensure that developing countries are actively involved in scientific production. Through a collaborative network, clinicians can more easily collect and compare data on clinical practices or patient populations in different countries. This can make international rehabilitation guidelines or neuromuscular treatment approaches more comprehensive.

The literature reports that increased international collaboration has a positive impact on the production of high‐quality outputs and the global visibility of research (H. Wang et al. 2022). Similarly, developing interdisciplinary or inter‐university networks in in‐country collaborations can increase research productivity and innovation (Tang et al. 2024). However, since this study is limited to reporting only the overall rate of co‐authorship, future research could broaden the scope of the investigation and analyze which countries have more intensive collaborations or between institutions within countries. This would provide more detailed insights into proprioception research's global reach and interaction networks.

However, this study does not assess intra‐country collaborations. Therefore, no specific data on the share of intra‐country institutional alliances can be provided.

### 4.6 Journal Productivity and Impact

According to Bradford's law, there were 25 core and 876 noncore journals. These core journals contained 33.66% of the total publications and constitute the main journals.

The most productive journals are the Experimental Brain Research, Journal of Neurophysiology, Vision Research, Journal of Neuroscience, Journal of Physical Therapy Science, Journal of Vision, Neuroscience Letters, Neuroscience, Frontiers in Human Neuroscience, and Journal of Sport Rehabilitation. However, the journals that had the highest impact were different. The Experimental Brain Research, Journal of Neurophysiology, Vision Research, Journal of Neuroscience, Journal of Physiology‐London, Journal of Athletic Training, Journal of Orthopaedic & Sports Physical Therapy, Archives of Physical Medicine and Rehabilitation, American Journal of Sports Medicine, and Neuropsychologia are the most influential journals according to their *h*‐indexes. The *h*‐index is more effective in predicting future success than other indicators (Hirsch 2007; Bozkurt and Bozkurt 2024). Since *h*‐index calculations typically consider the unique citation styles of each scientific discipline, comparing this index across disciplines may not be appropriate (Bozkurt and Bozkurt 2024). However, Experimental Brain Research ranked first among all bibliometric parameters and can thus be considered the most influential.

However, each of these indicators has its limitations. For example, the *h*‐index may not proportionally reflect individual contributions in multi‐authored publications. Because it is based on quantity rather than citation quality, it sometimes risks being overvalued (or inflated by self‐citations) in favor of “popular publications” (Bornmann and Daniel 2007). *g*‐index gives extra weight to highly cited publications but may not give a homogeneous result when compared across fields (Egghe 2006). The *m*‐index normalizes over the duration of the author's academic career but may not fully capture the duration of “activity” that the author wants to reflect, as it does not consider career breaks, multi‐disciplinary transitions, or industry changes (Hirsch 2005). Moreover, all three indicators are based on citation counts and do not directly reflect factors such as the qualitative dimension, clinical/practical impact, or originality of the research (Waltman 2016). Therefore, bibliometric indicators should not be used as a definitive “benchmarking tool” but rather as a supportive tool to track research trends in the field or provide insight into the overall level of impact (Kraus et al. 2022). This study's *h*‐index, *g*‐index, and *m*‐index results should be interpreted within this framework, together with data from other studies or additional criteria (e.g., publication quality ratings and expert assessments).

In addition, it is noteworthy that the American Journal of Sports Medicine has two articles on the list of the 10 most influential publications, further underscoring the journal's role in publishing and disseminating important research. Following these journals may be an effective way to stay updated on the latest research in proprioception. These findings could also assist researchers in choosing appropriate venues for publishing their work on proprioception‐related topics (Kaçmaz et al. 2024; Xu et al. 2024) because future discoveries in this field are more likely to be published in these journals (K. Wang et al. 2019). These journals highlighted the fields of neuroscience, physical therapy and rehabilitation, and vision. Research on proprioception has been published through interdisciplinary and multidisciplinary integration, suggesting the complexity of proprioception.

### 4.7 Strategic Diagrams

Strategic diagram and keyword trend analysis reveal proprioception research's historical and conceptual orientations. In the strategic diagram, Cluster 1 included balance, stroke, postural control, aging, and muscle spindle. This cluster represents the motor themes, so current and future studies will focus more on these themes (Ahmi 2021). These themes also have strong associations with other disciplines. Cluster 2 encompassed vision, human factors, motor control, motion, and multisensory integration. While these well‐developed themes tend to be isolated, showing weak connections between the disciplines. Cluster 3 included psychophysics, which appears to be a new research topic. Although there are few studies on this theme, their number is increasing. This area is still in its infancy, providing ample opportunity for development. Cluster 4 had the keywords rehabilitation, knee, range of motion, proprioceptive neuromuscular facilitation, and exercise. These topics have been well‐studied in the past. Thus, it is clear from this strategic diagram that rehabilitation‐centered research is currently the thematic group with the greatest prevalence and impact in proprioception. The 10 most influential articles include three that focus on the “knee.” The knee is among the most researched topics in sports rehabilitation, likely due to the high incidence of knee injuries, representing one of the largest clinical and public health burdens related to injuries (Prill et al. 2021). Similarly, exercise and range of motion are central to rehabilitation. Exercise is the primary tool, and improving range of motion is one of the most common goals in this process. Proprioceptive neuromuscular facilitation is another frequently utilized technique in rehabilitation (Silva et al. 2022). Therefore, it is clear that studies on proprioceptive neuromuscular facilitation and the knee have significantly contributed to the literature in the past.

### 4.8 Trending Topics

The main trending topics include “pain,” “postural balance,” “proprioceptive neuromuscular facilitation,” and “upper limb.” Pain is one of the most common reasons patients seek healthcare (Treede et al. 2019) and deteriorates proprioception and postural balance (Röijezon et al. 2015), which may be why these subjects are often studied with proprioception. Motion and muscle spindles exhibited the longest trending durations. Muscle spindles are recognized as the primary source of proprioception (Röijezon et al. 2015). Therefore, it is not unusual that many studies focus on the muscle spindles. These subjects' rise suggests that various research interests and relationships are becoming more well‐known, suggesting that these areas will likely attract increased attention.

### 4.9 Strategic Diagram and Keyword Trend Analysis

There are several reasons for the significant rise in the keyword “rehabilitation” in recent years. First, the increase in the aging population and chronic diseases has brought the need for neuromuscular rehabilitation to the forefront; accordingly, research on the strategic importance of proprioception in clinical practice has increased (Tang et al. 2024). Second, the increase in multidisciplinary approaches—for example, studies at the intersection of physiotherapy, geriatrics, sports sciences, and neuroscience—has led researchers to focus on this theme, emphasizing the role of proprioception more in rehabilitation processes (J.‐W. Chen et al. 2022). Third, the need for evidence‐based medical practice has encouraged quantitative analyses to measure the effectiveness of clinical rehabilitation protocols, rapidly popularizing the theme of “rehabilitation” in proprioception research.

In contrast, the relative decline of concepts such as “motion perception” or “motion” may be related to the shift in research focus from basic sensory physiology to clinical practice and treatment outcomes. While perceptual and basic neuroscientific mechanisms have received much attention in the past, in recent years, the predominance of studies focusing on functional recovery and patient care has led to a decline in the frequency of motion perception‐themed studies. Furthermore, the conceptual basis for motion perception may be partially established or dispersed to higher‐level specialties in the neuroscientific literature (Oguz et al. 2024). Therefore, this shift in research trends reflects the field's tendency to move away from “basic” mechanisms toward “applied” rehabilitation approaches.

Besides this, specific subfields, such as robotic proprioception or neuroplasticity, did not feature prominently in the trend data. Robotic proprioception has not become mainstream in research during this period despite offering strategies that can be integrated with wearable devices, AI‐enabled robotic systems, and prosthetic technologies (Xue et al. 2022). Similarly, studies directly related to neuroplasticity, such as the effects of proprioception on brain adaptation mechanisms, have been overshadowed by publications on rehabilitation and motor control (J.‐W. Chen et al. 2022). This suggests that while proprioception research is gaining weight in practical areas (e.g. clinical rehabilitation protocols, posture correction, sports injuries, etc.), it may not yet be sufficiently oriented toward using advanced technologies or in‐depth examination of neuroplastic change processes. Consequently, it's critical that future studies to address this gap in the existing literature by investigating the links between proprioception and robotic applications or neuroplasticity more comprehensively. Thus, the field's clinical, technological, and basic neuroscience aspects will be balanced.

### 4.10 Scientific Mapping

Keyword and co‐occurrence analyses offer an overview of the literature. The keywords have grown over time, likely due to increased scientific production. This heightened productivity may be linked to a greater emphasis on quantitative measurements in research, the promotion of evidence‐based healthcare, and the publish‐or‐perish phenomenon (Bartik et al. 2024).

The keywords “rehabilitation” and “balance” experienced the most significant breakthrough and became the most frequently used. The fact that the rehabilitation discipline emerged and developed relatively later than other medical professions may have contributed to these outcomes, which are comparable to the findings of Khatra et al. (2021).

The most commonly used keywords were rehabilitation, balance, stroke, knee, vision, postural control, range of motion, human, proprioceptive neuromuscular facilitation, and motor control, indicating that rehabilitation‐related keywords are at the forefront. Their prevalence demonstrates that rehabilitation is essential in proprioception research. In addition, stroke is one of the main reasons for disability and declines in movements, including range of motion, gait, postural control, and balance (Vecchio et al. 2024; Chiaramonte et al. 2024). Proprioceptive neuromuscular facilitation is a commonly used application that improves motor output and includes cutaneous, proprioceptive, and auditory stimulation. It can play a significant role in the rehabilitation of stroke and various other pathologies, which may be attributed to its prevalence, similar to the study of Xue et al. (2022) (Xu et al. 2024). In addition, the stage of stroke profoundly influences proprioceptive outcomes. For instance, patients in the subacute phase display distinct patterns of proprioceptive impairment compared with those in the chronic phase (Vecchio et al. 2024; Chiaramonte et al. 2024). Despite stroke being one of the most frequently examined conditions concerning proprioception, the literature on subacute stroke remains particularly scarce. This paucity of data limits our ability to capture the dynamic changes and potential recovery mechanisms unique to the early poststroke period. Consequently, no comprehensive framework integrates proprioceptive assessment and intervention strategies across different stages of stroke recovery. Future research should prioritize longitudinal investigations and the development of stage‐specific protocols to standardize outcome measures and tailor rehabilitative interventions accordingly.

### 4.11 The Co‐Occurrence Analysis

The co‐occurrence analysis identified two clusters. Within each cluster, a different discipline collaborates with neurophysiology/neuroscience studies, mainly on perception, and rehabilitation studies, mainly on physical function and performance. The clusters show the interdisciplinary nature of proprioception research. However, the literature does not have many interdisciplinary studies based on mutual collaboration; therefore, the connections between the clusters are weak.

These subjects offer insightful information about the topics and variables that attract research interest. First, neuroscience studies form the foundation for understanding proprioception. This field emphasizes the neurophysiological mechanisms and functions of proprioception within the central nervous system. This affirms that the cognitive and neural foundations of proprioception are closely linked to the fields of neurophysiology, particularly with perception. Second, the terms in the rehabilitation indicate that research focuses on peripheral neuromuscular mechanisms and function, evaluating afferent inputs and efferent outputs on physical function, performance, and various neuromuscular pathologies. As a result of the bibliometric analysis, proprioception is a key competence for the perception and precision of movements and is a significant topic in health sciences. Proprioception research is extensively studied as a central theme in rehabilitation research. However, studies on surgery and other invasive applications remain limited. Approaching proprioception through interdisciplinary means may foster a deeper understanding of the concept. By encouraging building on decades of previous research, publishing in specialist publications, collaborating across disciplines, and exploring under‐researched areas, investigators can enhance the impact and advancement of proprioception research (Xue et al. 2022; Ma et al. 2024). In future studies, multidisciplinary and interdisciplinary collaborations on proprioception, encompassing fields such as neuroscience, psychology, physical therapy, surgery, engineering, and robotics, should be significantly strengthened to facilitate comprehensive and collaborative research.

### 4.12 Bridging the Identified Research Gaps

Specific methodological approaches and interdisciplinary collaborations are crucial to filling the research gaps identified in this study. First, advanced neuroimaging (e.g., fMRI, MEG) and sensor‐based data collection (e.g., wearable technologies, accelerometers) methods may allow for a more detailed investigation of proprioception processes at both central and peripheral levels (Tang et al. 2024). Second, AI and machine learning‐assisted analyses can integrate multidimensional data (physiological signals, biomechanical measurements, and clinical assessments) and may open new horizons in detecting interindividual differences (Xue et al. 2022). Third, collaborative projects between different fields, such as rehabilitation sciences, neuroscience, engineering, psychology, and sports sciences, will strengthen the technical infrastructure and accelerate the evolution of proprioception research into innovative applications by bringing together theoretical approaches from different perspectives (J.‐W. Chen et al. 2022). Finally, quantitative data combined with mixed methods designs or qualitative research (e.g. in‐depth interviews, focus groups) can allow for clinically meaningful results and a closer focus on the patient/participant experience (Kraus et al. 2022). These proposed directions will play a critical role in closing the gaps in proprioception and creating a more holistic, multidimensional research ecosystem in the long term.

### 4.13 Comparison With Related Research Fields

Although the main focus of this study was to examine trends in proprioception research in depth, no comparison with bibliometric trends in adjacent fields such as motor control or sensory processing was made. However, such a comparison could have more clearly revealed the unique developmental dynamics of proprioception research and the common methodological/theoretical approaches it shares with different disciplines (J.‐W. Chen et al. 2022). For example, it is known that robotic applications and AI‐supported analysis have become widespread in the motor control literature in recent years (Xue et al. 2022). However, the extent to which similar technological adaptations have or have not been adapted to proprioception studies could be more comprehensively determined through a comparative bibliometric analysis. Likewise, basic neurophysiological approaches in sensory processing can provide valuable clues to explain how mechanisms related to proprioception are integrated into a broader sensory framework (Tang et al. 2024).

### 4.14 Implications for Researchers, Clinicians, and Policymakers

This bibliometric research reveals that proprioception has grown significantly in recent years, particularly in rehabilitation, neurophysiology, and posture control. However, for a more comprehensive progress and effective contribution to science, attention should be paid to the following points:

Researchers: It is recommended that the theoretical framework be deepened by investigating under‐studied topics in the existing literature (e.g., psychophysics, robotic proprioception, AI‐assisted modeling). Future research could examine the scientific productivity, citation dynamics, and thematic orientations of the field in a broader context by comparing the field of proprioception with both motor control and various sensory processes literature using quantitative (bibliometric) and qualitative (e.g., expert opinions, field studies) methods. In this way, it will be possible to see more comprehensively how proprioception studies interact with different disciplines and to identify new opportunities for future research collaborations.

Furthermore, fostering interdisciplinary collaborations—with fields such as engineering, sports science, neuroscience, and psychology—will allow learning and developing new methodological approaches and collecting data from more holistic perspectives (Xue et al. 2022).

Clinicians: Findings suggest that proprioception is becoming a central element of clinical rehabilitation. Clinicians could benefit from these advancements by incorporating cutting‐edge techniques into rehabilitation practices, especially those involving neuroprosthetics and AI‐driven models. Incorporating innovative techniques to enrich daily practice, especially in stroke, neurodegenerative diseases, and orthopedic injuries, could significantly contribute to patients' functional recovery (J.‐W. Chen et al. 2022).

Policymakers: Geographical distribution is still uneven, with many regions (Asia, Africa, South America, etc.) underrepresented. Diversification of research funding and international cooperation programs could lead to more inclusive work globally. This would enhance the literature and facilitate to a greater understanding of cultural and demographic differences. Policymakers should also consider investing in research that fosters global collaboration, advances technological innovations in rehabilitation, and ensures the inclusion of diverse populations in future studies to meet the changing needs of individuals affected by proprioception disorders.

### 4.15 Study Limitations

Acknowledging several limitations in this bibliometric study and the bibliometric limitations discussed above is crucial. Since bibliometric analyses provide a statistical and global perspective, examining the content of each study on proprioception and its contribution to the field is challenging. Furthermore, this study is based solely on data from the WoS, which may exclude valuable research published in other databases. This may lead to citation biases and limit the comprehensiveness of the study. However, merging data sets with different formats and orders results in technical issues, including data loss and overlap, which diminish the reliability of the outcomes. Therefore, it is advisable to focus on a single database. In addition, excluding non‐English studies may have omitted important findings from international research communities. These limitations underscore the need for further bibliometric analyses incorporating more diverse data sources and languages.

## 5 Conclusion

In conclusion, proprioception research is a vibrant, developing field with much promise for growth. This bibliometric analysis summarizes the past and present development, prominent themes, and global impact of proprioception research. The findings show that the field focuses on rehabilitation, neurophysiology, and posture control, while multidisciplinary approaches and advanced technologies offer untapped potential. As proprioception grows in clinical and basic research, new technologies, interdisciplinary collaborations, and inclusive research policies can lead to more in‐depth and effective applications. Researchers, clinicians, and policymakers must work together to prioritize these areas and foster the next generation of proprioception research to enhance treatment options and improve patient outcomes.

Focus on Under‐Studied Topics: It is recommended that themes with a relatively limited number of studies be further investigated, such as psychophysics, robotic proprioception, and AI‐enabled applications. In addition, developing robotic rehabilitation, wearable sensor systems, and augmented/virtual reality applications will shed light on both the neural and functional aspects of proprioception, enabling a comprehensive examination of motor learning and neuroplasticity processes and shaping the future trajectory of proprioception research. In the coming period, innovative technologies such as AI‐assisted proprioceptive modeling, neuroprosthetics, and human‐machine interfaces are expected to significantly transform the study of proprioception at the basic science and clinical practice level. Integrating multidimensional data can predict interindividual changes more accurately by offering more precise modeling of proprioceptive signals. Likewise, advances in the field of neuroprosthetics are paving the way for both the design of devices that support functional recovery in neurological conditions such as spinal cord injury or stroke and more natural and effective prosthetic control for individuals with limb loss.

Development of Interdisciplinary Collaborations: We recommended that proprioception research be carried out in a multidisciplinary framework by conducting joint projects with engineering, neuroscience, sports sciences, psychology, and rehabilitation. Integrating different data collection and analysis methods may obtain more comprehensive and innovative results. Future studies integrating these innovative technologies into research protocols by establishing multidisciplinary teams and international collaboration networks will contribute greatly to proprioception's scientific advancement and field applications. This will make theoretical knowledge in the field and effective and personalized treatment methods that prioritize patient and community benefit possible.

Advanced Methodological Approaches: Advanced neuroimaging (fMRI, MEG) and sensor‐based applications (e.g., wearable technologies) can further investigate proprioceptive feedback mechanisms at both central and peripheral levels. Approaches like mixed methods designs or big data analytics may better reflect the clinical impact and individual differences.

Increasing Geographic and Cultural Diversity: One of the significant limitations identified in this study is the underrepresentation of research from diverse geographical regions. Since most publications originate from North America and Europe, future studies should encourage collaborations across regions to account for cultural, environmental, and demographic differences in proprioception mechanisms. Expanding the geographic scope of research will provide a more comprehensive understanding of proprioception and its variations across populations, especially in underrepresented regions such as Asia, Africa, and Latin America.

Translation to the Clinic: The findings may guide the development of specific exercise protocols or technology‐based interventions to support proprioception in different patient groups (stroke, degenerative diseases, sports injuries, etc.). New research results should be interpreted to improve patient care by directly incorporating them into clinical guidelines.

Proprioception research can be enriched in quantity, quality, and impact in line with these recommendations. Combining an interdisciplinary perspective and advanced methodological tools will make both theoretical deepening and innovative practice solutions possible across the field.

## Author Contributions

**Kevser Sevik Kacmaz**: Conceptualization, investigation, writing – original draft, writing – review and editing, visualization, methodology, validation, project administration, data curation, software, formal analysis.

## Conflicts of Interest

The author declares no conflicts of interest.

## Peer Review

The peer review history for this article is available at https://publons.com/publon/10.1002/brb3.70610

## Data Availability

The data that support the findings of this study are available on request from the corresponding author. The data are not publicly available due to privacy or ethical restrictions.
